# New Genetic Insights from Autoimmune Thyroid Disease

**DOI:** 10.1155/2012/623852

**Published:** 2012-02-28

**Authors:** Terry F. Davies, Rauf Latif, Xiaoming Yin

**Affiliations:** Thyroid Research Unit, Mount Sinai School of Medicine, James J. Peters VA Medical Center, New York, NY 10468, USA

## Abstract

The autoimmune thyroid diseases (AITDs) (Graves' disease and Hashimoto's thyroiditis) are complex genetic diseases which most likely have more than 20 genes contributing to the clinical phenotypes. To date, the genes known to be contributing fall into two categories: immune regulatory genes (including *HLA, CTLA4, PTPN22, CD40, CD25, and FCRL3*) and thyroid-specific genes (*TG and TSHR*). However, none of these genes contribute more than a 4-fold increase in risk of developing one of these diseases, and none of the polymorphisms discovered is essential for disease development. Hence, it appears that a variety of different gene interactions can combine to cause the same clinical disease pattern, but the contributing genes may differ from patient to patient and from population to population. Furthermore, this possible mechanism leaves open the powerful influence of the environment and epigenetic modifications of gene expression. For the clinician, this means that genetic profiling of such patients is unlikely to be fruitful in the near future.

## 1. Introduction

Many diseases have a tendency to run in families, and we know that this may be due to either environmental influences, or family genetics, or both. The autoimmune thyroid diseases (AITDs), Graves' disease and Hashimoto's thyroiditis, are typical examples of such complex diseases and have been recognized for many years as having an important genetic component. In the last 10 years we have learned many new insights into the way genetic influences can enhance thyroid autoimmunity, but there remain large gaps in our knowledge which are unlikely to be filled without major theoretical and technical advances. This brief review examines the current state of knowledge and what new insights we have gained from exploring the genetics of the AITDs, and in particular Graves' disease.

## 2. Thyroid Autoantibodies

Autoantibodies to thyroid peroxidase (TPO) and thyroglobulin (Tg) are reflections of thyroid disease rather than causative agents [1]. Hence, such thyroid autoantibodies may develop before the onset of clinical AITD and have been long known to increase the risk of developing clinical AITD [2]. The recognition of a familial association for the production of thyroid antibodies [3] led to studies of first-degree relatives of probands with AITD and indicated a dominant pattern of inheritance. Indeed, up to 50% of the siblings of patients with AITD are thyroid antibody positive [4, 5] in contrast to ~15% in the general population [6]. Several segregation analyses have also shown a Mendelian dominant pattern of inheritance for the expression of thyroid autoantibodies [7, 8], and genetic transmission of TPO antibody subclass “fingerprints” has suggested that the pattern of autoantibody recognition of the TPO antigen was also genetically transmitted [9].

## 3. Genetic Susceptibility to AITD

The recognition of an association between AITD and certain human leukocyte antigens (HLA) first provided a mechanism for the genetic contribution to Graves' disease and Hashimoto's thyroiditis [10]. This association has been especially well seen in identical twins [11]. The HLA antigens provide a means for the immune system to recognize thyroid antigenic peptides, and recent data have demonstrated this enhanced association as secondary to the presence of particular residues in the HLA class II binding pocket such as Arg 74 [12]. In addition, as the pathological and molecular mechanisms involved in AITD became known, many of which were not only common to all autoimmune diseases but also highly variable between individuals; this allowed the recognition of candidate genes responsible for disease susceptibility. Such genes could then be assessed by either linkage analysis or association studies (see Table 1).

## 4. Detecting Susceptibility Genes in AITD

The candidate HLA gene complex was first associated with AITD in association studies but then failed to show linkage with AITD [14]. This showed that the genetic contribution of HLA to AITD was not strong enough to be seen in linkage analyses [13]. This indicated that association studies were more likely to detect genes contributing small effects on disease susceptibility. As a consequence of the Human Genome Project, it became possible to identify genes for diseases that had a complex genetic basis without resorting to the candidate gene approach. This was achieved by “typing” individuals using a genome screen of genetic markers, at first with microsatellites (1 microsatellite per 10 cM DNA) and later single-nucleotide polymorphisms (SNPs) (~1 SNP per < 1 cM DNA), which covered the entire genome (Table 2) [15]. Then investigators observed which markers segregated with the disease. However, the reduced sensitivity of linkage analyses, compared to association studies, made it more difficult to perform these analyses for the complex traits characteristic of a non-Mendelian pattern of inheritance and with variable clinical phenotypes. However, using large numbers of SNPs, developed as a result of the HapMap project [16, 17], and which had a much greater degree of coverage of the whole genome, it was easier to decipher which markers segregated with the disease using association analyses. These SNP markers occur more frequently than microsatellite markers and are easy to detect, allowing for greater genetic sensitivity. The suspected gene region can then be further narrowed with more dense SNPs and the gene can be identified. Results are now available for a variety of autoimmune diseases including rheumatoid arthritis and type 1 diabetes mellitus [18] and most recently for AITD [19].

It is obviously essential that whole-genome association study results must be reliably and repeatedly reproduced, but the complexity of this type of analysis and the high cost have raised problems [20, 21]. If common diseases are associated with common risks, then replication across populations can be expected. But common diseases may be related to population-specific risks, and, therefore, such data can only be reproduced in the same population as that which was studied in the original report. Reproducibility had been a problem for studies that used microsatellite screening, including the studies in patients with AITD, and this problem has persisted in the much larger studies using whole-genome association studies such as in those analyzing Parkinson's disease and also obesity. Hence, all reports of genetic linkage and association require confirmation by independent studies before they can be accepted.

## 5. Genes for AITD

The *HLA* and *CTLA4* genes were the first genes identified by the candidate approach [22, 23] (Table 3).

 As discussed earlier, the *HLA* genes make up the major histocompatibility complex (MHC) which contains many genes related to immune system function in humans. These include HLA class I (A, B, and C), HLA class II (DP, DM, DOA, DOB, DQ, and DR), and HLA class III (coding for other immune proteins). The major GD-associated *HLA*, *HLA-DR3*, locates at the *HLA DR* locus and plays a key role in the normal immune response by binding peptide antigens and presenting them to T-cell receptors.

 The cytotoxic T-lymphocyte-associated protein 4 (*CTLA4*) gene is an immune regulatory molecule, which is expressed on the surface of Helper T cells and transmits an inhibitory signal to T cells. In addition to the *HLA* and *CTLA4* gene loci, there are confirmed associations (2 or more reports) for a number of genes also common to many autoimmune diseases: *PTPN22*, *CD40*, *IL2RA* (*CD25*), and *FCRL3 *(Table 3). 

 The gene for protein tyrosine phosphatase, non-receptor type 22 (lymphoid), also known as just *PTPN22*, encodes a protein tyrosine phosphatase expressed primarily in lymphoid tissues. This enzyme associates with the molecular adapter protein CBL and may be involved in regulating CBL function in the T-cell receptor signaling pathway. A variant of the *PTPN22 *encodes Lyp phosphatase (Lyp620W) and confers risk for multiple autoimmune diseases. Most recently, Zhang et al. [24] reported that levels of the Lyp620W variant were decreased in human T and B cells, and its calpain binding and cleavage were increased relative to wild-type Lyp620R. Therefore, calpain-mediated degradation with consequently reduced Lyp expression and lymphocyte and dendritic cell hyperresponsiveness represents a potential mechanism for unregulated autoimmunity. The LypR620W variant, with an arginine to tryptophan substitution, loses its function and influence on immune responses, which increases the risk for autoimmune disease.

 The *CD40* molecule, or TNF receptor superfamily member 5 gene, encodes a costimulatory receptor which is essential in mediating a broad variety of immune and inflammatory responses including T-cell-dependent immunoglobulin class switching, memory B-cell development, and germinal center formation [25]. The interleukin 2 (IL2) receptor alpha gene (*IL2RA* or *CD25*) encodes one of the subunits of the IL-2 receptor that binds IL-2 and is vital in the regulation of T-cell function. The Fc receptor-like protein 3 (*FCRL3*) gene encodes a protein containing an immunoreceptor-tyrosine activation motif and immunoreceptor-tyrosine inhibitory motif in its cytoplasmic domain and may play a role in immune regulation.

To date, the only thyroid-related genes associated with AITD are *TG* (the gene encoding thyroglobulin) [26], in both Graves' disease and Hashimoto's thyroiditis, and *TSHR* (the gene encoding the thyrotropin receptor) restricted to Graves' disease [27, 28] (Table 3).

The thyroglobulin (*TG*) gene encodes a large glycoprotein homodimer produced exclusively by the thyroid gland. It acts as a substrate for the synthesis of thyroid hormones thyroxine (T4) and triiodothyronine (T3) as well as the storage of the inactive forms of thyroid hormone and iodine. How this gene influences susceptibility is unclear but Stefan et al. [29] have recently described a genetic/epigenetic mechanism by which a newly identified *TG* promoter SNP variant predisposes to AITD. Sequencing analyses followed by case control and family-based association studies identified a SNP (−1623A→G) that was associated with AITD in the Caucasian population, and the associated nucleotide substitution SNP (−1623A/G) modified a binding site for interferon regulatory factor-1 (IRF-1), a major interferon-induced transcription factor, indicating enhanced sensitivity to this inflammatory cytokine [29].

The thyroid stimulating hormone receptor (*TSHR*) gene encodes a membrane protein that signals through binding TSH ligand and is a major controller of thyroid cell growth and metabolism. SNPs in intron 1 (in Caucasians) and intron 7 (in Japanese) have been associated with Graves' disease in a number of studies [27, 28, 30]. Recent data suggest that TSHR-associated SNPs are related to defective thymic tolerance for the TSHR as shown by reduced expression within the thymus gland where it is needed to delete TSHR autoreactive T cells [31].

 Because all the identified susceptibility genes found to date appear to have a low level of contribution to genetic susceptibility, a number of whole-genome screening studies have also been attempted in AITD to find more important genes [32–36]. One whole-genome association study using only 10^4^ nonsynonymous SNPs (those involving parts of a gene likely to affect the product character) showed a number of the previously recognized genes, as well as locating some new sites, but the new sites could not subsequently be confirmed [37, 38]. Most recently, the first full genome-wide study of Graves' disease with 660 K SNPs has now been reported from China [19]. This study again identified many of the known genes for AITD, but also described two new sites on chromosomes 6q and 4p. These await further confirmation. Again, however, no very highly associated new genes have emerged.

## 6. The Degree of Enhanced Susceptibility Remains Low

All the genes associated with AITD are individually able to confer only modest degrees of disease susceptibility (expressed as odds ratios, see Table 3). Hence, these data only allow us to conclude that the AITDs, both Graves' disease (including Graves' ophthalmopathy) and Hashimoto's thyroiditis, are complex genetic disorders involving multiple genes that may interact to provide a susceptible background for disease development. Furthermore, there appear to be disease-specific genes, such as the gene encoding the TSHR in Graves' disease and a larger group of susceptibility genes, such as *CTLA4*, which are common to many autoimmune diseases. This combination of gene polymorphisms likely allows epigenetic phenomena, subsequent to a variety of influences such as infection and the environment, to initiate disease.

## 7. The Controversy over Major Genes in AITD

After the clarification that multiple genes are at work in AITD, it is likely that more than 20 potential genes contribute to the AITD phenotypes. But major genes, those essential to disease development, have not been found [39]. A major gene should be involved in the majority of patients with the disease, and the risk ratios, even for HLA, do not reveal such a gene (Table 3). This most likely means that different combinations of genes may produce similar clinical phenotypes or that epigenetic phenomena are dominant. So far, in the whole-genome screening of families, siblings, and populations with AITD, a number of sites have been established for Graves' disease and Hashimoto's thyroiditis susceptibility, but none of them have had very high statistical values (LOD scores) [32, 33, 35]. This finding has been true not just for AITD, but also for other autoimmune diseases including type 1 diabetes mellitus. This is best understood by thinking of HLA once again. Not every patient with Graves' disease has the associated HLA-DR3 subtype and not even the associated Arg74 in its binding pocket, irrespective of the HLA-DR subtype [12]. Hence, the disease can occur in the absence of the expected HLA association.

## 8. A Note on Epigenetics

One mechanism by which environmental factors may combine with genetic risk to promote AITD is by altering the epigenetic control of gene expression as seen, for example, in the pancreas [40] and as shown for a virus interacting with a susceptibility gene in Crohn's disease [41]. While little is known about such interactions with AITD, there has been wide confirmation of a role for X chromosome inactivation (XCI) [42, 43]. Patients with AITD more often than expected showed a biased expression of a maternal or paternal X chromosome leading to the hypothesis that the poorly expressed chromosome could become active in certain tissues such as the thyroid and express new antigenic sequences not previously recognized by the immune system. These potential mechanisms for enhanced susceptibility to AITD require further exploration.

## 9. Conclusions

How environmental factors combine with genetic risk at the molecular level to promote complex genetic diseases such as AITD is largely unknown. The genes that are linked to and/or associated with AITD are each small contributors to genetic risk. Multiple-gene polymorphisms (combinations of haplotypes) appear to be needed to develop AITD and may differ between geographic populations secondary to epigenetic influences. Much remains to be learned.

## Figures and Tables

**Table 1:** Methods of genetic analysis.

(A) *Linkage analysis *	

This is based on the principle that the chance for a recombination event between 2 loci (i.e., a marker, such as the candidate gene, and the true disease gene) is proportional to the chromosomal distance between them. Therefore, if a marker is close to a disease susceptibility gene, this marker will cosegregate with the disease in families.	
The logarithm of odds (LOD) score is a measure of the evidence for or against linkage between a marker and a trait or disease [13]. LOD score analysis has had important advantages for the study of AITD because it has allowed a way to test for the presence of heterogeneity within the data set and allowed deduction of the mode of inheritance and the degree of penetrance from the linkage data.	
Linkage studies are highly specific but have been clearly shown not to be highly sensitive.	

(B) *Association studies *	

These studies simply compare the presence of a disease marker (such as the candidate gene) in the disease population with the presence of the marker in a control population without the disease.	
Here, the difficulty may lie in the appropriate control population, which needs to be comparable and large.	
If this difficulty is overcome, association studies can reveal a genetic influence, and with large patient groups, this type of study can be highly sensitive.	

**Table 2:** Methods for whole-genome screening.

(A) *Microsatellites *	

These are regions in the genome that are composed of repetitive sequences. The most common microsatellites are the CA (dC-dA)n repeats. Microsatellite loci are highly polymorphic because of variation in the number of repeats (usually there are 5 to 15 alleles per locus), and they are uniformly distributed throughout the genome at distances of fewer than 1 million base pairs [15]. Therefore, microsatellites served as useful markers in linkage studies designed to search for unknown disease susceptibility genes. Investigators then further narrowed the suspected gene region with more dense markers, and the gene could be identified.	

(B) *Single-nucleotide polymorphisms* (SNPs)	

Without having to enlist families, it is now possible to use genome-wide association studies involving up to 10^6^ SNPs (on a microchip), each of which is in linkage disequilibrium with large segments of the genome, and then analyze their association with any disease.	

**Table 3:** Genes linked and/or associated with autoimmune thyroid disease.

Gene symbol	Gene name	Chromosome location	Odds ratio
*HLA*	Major histocompatibility complex	6p21	2.0–4.0
*CTLA4*	Cytotoxic T-lymphocyte-associated protein 4	2q33	1.5–2.2
*PTPN22*	Protein tyrosine phosphatase, non-receptor type 22 (lymphoid)	1p13	1.4–1.9
*CD40*	CD40 molecule, TNF receptor superfamily member 5	20q11	1.3–1.8
*IL2RA* (*CD25*)	Interleukin 2 receptor, alpha	10p15	1.1–1.4
*FCRL3*	Fc receptor-like 3	1q23	1.1–1.3
*TG*	Thyroglobulin	8q24	1.3–1.6
*TSHR*	Thyroid-stimulating hormone receptor	14q31	1.4–2.6

## Abbreviations

AITD: Autoimmune thyroid disease
LOD: Logarithm of odds
SNP: Single-nucleotide polymorphism.
